# Longitudinal Speech Recognition in Noise in Children: Effects of Hearing Status and Vocabulary

**DOI:** 10.3389/fpsyg.2019.02421

**Published:** 2019-10-25

**Authors:** Elizabeth A. Walker, Caitlin Sapp, Jacob J. Oleson, Ryan W. McCreery

**Affiliations:** ^1^Pediatric Audiology Laboratory, Department of Communication Sciences and Disorders, University of Iowa, Iowa City, IA, United States; ^2^Department of Biostatistics, University of Iowa, Iowa City, IA, United States; ^3^Center for Hearing Research, Audibility, Perception, and Cognition Laboratory, Boys Town National Research Hospital, Omaha, NE, United States

**Keywords:** children, vocabulary, working memory, hearing loss, speech recognition

## Abstract

**Objectives:** The aims of the current study were: (1) to compare growth trajectories of speech recognition in noise for children with normal hearing (CNH) and children who are hard of hearing (CHH) and (2) to determine the effects of auditory access, vocabulary size, and working memory on growth trajectories of speech recognition in noise in CHH.

**Design:** Participants included 290 children enrolled in a longitudinal study. Children received a comprehensive battery of measures annually, including speech recognition in noise, vocabulary, and working memory. We collected measures of unaided and aided hearing and daily hearing aid (HA) use to quantify aided auditory experience (i.e., HA dosage). We used a longitudinal regression framework to examine the trajectories of speech recognition in noise in CNH and CHH. To determine factors that were associated with growth trajectories for CHH, we used a longitudinal regression model in which the dependent variable was speech recognition in noise scores, and the independent variables were grade, maternal education level, age at confirmation of hearing loss, vocabulary scores, working memory scores, and HA dosage.

**Results:** We found a significant effect of grade and hearing status. Older children and CNH showed stronger speech recognition in noise scores compared to younger children and CHH. The growth trajectories for both groups were parallel over time. For CHH, older age, stronger vocabulary skills, and greater average HA dosage supported speech recognition in noise.

**Conclusion:** The current study is among the first to compare developmental growth rates in speech recognition for CHH and CNH. CHH demonstrated persistent deficits in speech recognition in noise out to age 11, with no evidence of convergence or divergence between groups. These trends highlight the need to provide support for children with all degrees of hearing loss in the academic setting as they transition into secondary grades. The results also elucidate factors that influence growth trajectories for speech recognition in noise for children; stronger vocabulary skills and higher HA dosage supported speech recognition in degraded situations. This knowledge helps us to develop a more comprehensive model of spoken word recognition in children.

## Introduction

Every year, approximately three in 1,000 children are born with a significant hearing loss (Mehra et al., 2009). Children who are hard of hearing (CHH) have sufficient residual hearing to benefit from amplification. With the advent of newborn hearing screening, they are now being identified and fitted with hearing aids (HAs) during infancy (Holte et al., 2012). Early access to technology and services is posited to have a positive, long-term impact on functional outcomes, which results in the vast majority of CHH being educated in regular education settings (Page et al., 2018). As most CHH rely entirely on spoken language to communicate, they face significant challenges as they enter classrooms that are likely to have poor acoustics (Knecht et al., 2002). Most academic and extracurricular settings are characterized by background noise, which negatively affects speech recognition and academic outcomes in children with normal hearing (CNH), and has even greater consequences for CHH. Even though CHH have documented weaknesses with listening in noise (Crandell, 1993; Uhler et al., 2011; Caldwell and Nittrouer, 2013; Leibold et al., 2013; McCreery et al., 2015; Klein et al., 2017; Ching et al., 2018), there is little research on how their ability to recognize speech in noise develops over time during the school-age years. Increased knowledge in this area impacts both clinical decision-making and theoretical understanding of the mechanisms that drive listening in noise. The goals of the current study are twofold: (1) to investigate growth rates in speech recognition in noise for school-age CHH and CNH, and (2) to investigate the impact of auditory access and cognitive-linguistic abilities on CHH’s ability to listen in adverse acoustic conditions over time.

Given their reduced access to spectral and temporal cues in the speech signal, as well as reduced binaural processing, it is not surprising that listening in noise is a challenge for CHH. McCreery et al. (2015) examined word and phoneme recognition in noise in 7- to 9-year-old CHH and age-matched CNH. Even with amplification, CHH rarely reached the same level of performance as CNH in noise. Caldwell and Nittrouer et al. (2013) evaluated kindergartners with normal hearing, HAs, or cochlear implants (CIs) on measures of speech recognition in quiet and in noise and found significant group differences in favor of CNH. The question that then arises is whether children with hearing loss can eventually catch up with their peers, if the gap in speech recognition in noise widens over time, or if they show persistent but stable deficits in recognizing speech in noise. Given that adults with hearing loss show difficulties with listening in noise (Dubno, 2015), we would predict that CHH will not show speech recognition scores that are commensurate with CNH. On the other hand, CNH might reach a floor level on speech recognition in noise tasks, allowing CHH to eventually close the gap. It also seems improbable that the gap in speech recognition would widen over time; however, a recent study by Walker et al. (2019) indicated an increasing gap with age between CHH and CNH in identifying words during a gating paradigm. The third option, parallel growth rates between CHH and CHH, would appear to be the most reasonable prediction given what we know from previous research. This hypothesis has not been tested empirically, however, because much of the research to date is cross-sectional or has too few subjects or data points to conduct longitudinal analyses. Thus, there is minimal knowledge about the developmental aspects of speech recognition for CHH compared to CNH, or the cognitive and peripheral factors that support growth in listening skills over time. The question of developmental trajectories in speech recognition in noise can only be effectively addressed with longitudinal data sets, which are lacking in the research literature on CHH.

In addition to limited longitudinal data, previous large-scale studies of speech recognition in children with hearing loss have focused primarily on children with congenital, severe-profound hearing loss who use CIs (Davidson et al., 2011; Robinson et al., 2012; Ching et al., 2014; Dunn et al., 2014; Easwar et al., 2018). CHH are either excluded from these research studies or combined with children who are deaf, making it difficult to isolate the effects of mild to severe hearing loss on speech recognition. The studies that have been conducted with CHH have some limitations. First, children have been tested with words in quiet, rather than word or sentence recognition in noise (Stiles et al., 2012). Identifying monosyllabic words in quiet is not representative of the everyday listening experiences of children (Magimairaj et al., 2018) and may restrict individual differences for CHH, as many of these children will perform at or near ceiling levels (McCreery et al., 2015). Furthermore, speech recognition testing with background noise more accurately reflects listening experiences in realistic settings than monosyllabic word recognition in quiet (Kirk et al., 2012; Hillock-Dunn et al., 2014). Monosyllabic word recognition in quiet has minimal cognitive and linguistic processing demands, which are required in real-world listening environments (Walker et al., 2019). A second limitation of the prior research is that the focus is often on the influence of age at confirmation of hearing loss or age at amplification on speech recognition in noise (Sininger et al., 2010; Ching et al., 2013). Although it is important to evaluate the effectiveness of early hearing detection and intervention services, it is also important to understand the combined effects of auditory access, cognitive and linguistic abilities on listening development. There has been a great deal of attention directed toward understanding speech recognition skills in children with hearing loss, but we still lack a clear understanding of the mechanisms that drive developmental growth.

In environments with degraded signals (either due to poor acoustics or reduced hearing levels), listeners rely on higher level cognitive and linguistic skills to interpret information about the input (Nittrouer and Boothroyd, 1990). According to the Ease of Language Understanding (ELU) model (Rönnberg et al., 2013), adults with higher cognitive skills compensate in listening situations with distorted or missing information because they can use their memory and linguistic skills to repair the distorted signal (Akeroyd, 2008; Rönnberg et al., 2008; Tun et al., 2010; Zekveld et al., 2011). The findings supporting the predictions of the ELU model in children are mixed. Lalonde and Holt (2014) reported that parent report measures of working memory were positively correlated with speech recognition in quiet with 2-year-old CNH. McCreery et al. (2017) evaluated monosyllabic word and sentence recognition in noise for 96 5- to 12-year-old CNH. Children with higher working memory skills (measured as a combination of complex visual and verbal working memory span scores) had better speech recognition in noise skills than children with lower working memory. On the other hand, there are several studies that do not support the predictions of the ELU model in children. Eisenberg et al. (2000) did not find an association between working memory capacity (measured with forward digit span) and spectrally degraded speech recognition in CNH after controlling for age. Magimairaj et al. (2018) also did not find that working memory capacity (measured with forward digit span, auditory working memory, and complex working memory span tasks) was predictive of speech recognition in noise for 7- to 11-year-old CNH. The differences in findings may be due to the predictor variables and/or the outcome measures. Eisenberg et al. used a short-term working memory test (i.e., storage only), as opposed to complex working memory span measures (i.e., storage and processing). The proponents of the ELU model have posited that simple span tests like digit span are not good predictors of speech recognition (Rönnberg et al., 2013). McCreery et al. used sentences with no semantic context (which increased the memory load), whereas Magimairaj et al. used the Bamford-Kowal-Bench Speech in Noise sentences (BKB-SIN; Bench et al., 1979) which include semantic cues. It is also important to note that the effects of complex working memory span have not been thoroughly explored in CHH. More studies are needed to disentangle the associations between working memory, language, auditory access, and speech recognition in noise for children with hearing loss, who are most impacted by degraded acoustic input.

In addition to exploring the role of working memory capacity, the ELU model also predicts that language abilities will influence the ability to recognize degraded speech (Zekveld et al., 2011). Performance on sentence repetition tasks (which are used to measure speech recognition in noise) is likely tied to oral language skills (Klem et al., 2015). Stronger language skills allow individuals to make better predictions about an incoming message, even in the presence of limited sensory input (Nittrouer et al., 2013). Vocabulary knowledge accounts for a significant proportion of variance in word and sentence recognition in quiet for children with CIs and/or HAs (Blamey et al., 2001; Caldwell and Nittrouer, 2013), and language skills are significant predictors of speech recognition in noise for school-age CHH (McCreery et al., 2015; Klein et al., 2017; Ching et al., 2018). None of these studies included longitudinal data, so it was not possible to determine how these underlying mechanisms influence developmental trajectories of speech recognition in noise. In contrast to the former studies, Magimairaj et al. (2018) did not find that language skills were related to BKB-SIN scores, which they interpreted as an indication that speech recognition in noise is dissociated from language on that clinical measure. They did not include CHH as participants, however, and their language metric was a combined measure of receptive and expressive vocabulary, language comprehension, sentence recall, and inference-making. Thus, their composite language measure may have lacked sensitivity and masked variability, resulting in their reported finding of a dissociation between language and speech recognition in noise.

A third relevant factor to consider when examining sources of variance in speech recognition in noise is auditory access, particularly because CHH show large individual differences in this variable (McCreery et al., 2013; Walker et al., 2013). Auditory access has been explored as a predictor in several ways. One method is to use degree of hearing loss (i.e., pure tone average; PTA) as a predictor. Blamey et al. (2001) found that lower PTA was associated with better speech recognition in noise in children with moderate to profound hearing loss. In contrast, Sininger et al. (2010) examined auditory outcomes in young children with mild to profound hearing loss and found that PTA did not contribute to speech recognition skills. These mixed results may be related to the fact that PTA does not capture the everyday aided listening experiences of CHH. Because PTA measures only unaided audibility for very soft sounds, it does not reflect a child’s access to supra-threshold speech while wearing HAs.

An alternative to relying on PTA is to examine audibility levels, as measured by the Speech Intelligibility Index (SII). SII is a measurement that describes the proportion of speech accessible to the listener, with or without HAs. It accounts for the configuration of hearing loss, differences in ear canal size, and amplification characteristics of HAs. Studies have shown an association between SII and speech recognition in CHH (Stelmachowicz et al., 2000; Davidson and Skinner, 2006; Scollie, 2008; Stiles et al., 2012; McCreery et al., 2017); however, Ching et al. (2018) reported that aided SII did not contribute any additional variance to speech recognition in noise for 5-year-old CHH, after controlling for unaided hearing thresholds, non-verbal intelligence, and language skills. Children with HAs in the Ching et al. study were fitted within 3 dB of HA prescriptive targets, which likely reduced variability in SII.

A third way to examine auditory access is to consider individual differences in the amount of daily HA use. Only a few studies have looked at hours of HA use as a predictor variable of speech recognition in noise. McCreery et al. (2015) found that children with more hours of HA use showed higher scores on parent report measures of auditory skills and word recognition in quiet for toddlers and preschoolers with hearing loss. In contrast, Klein et al. (2017) did not find an effect of HA use on word and nonword recognition in school-age CHH. They acknowledged, however, that there was little variability in this factor among the participants, who were mostly consistent HA users.

To better understand the impact of auditory access on listening in noise, we propose to conceptualize the auditory experience of CHH as a combination of unaided hearing, aided SII, and amount of HA use (Walker et al., accepted). Our past studies showed that CHH demonstrate large individual differences in aided audibility (McCreery et al., 2013) and amount of HA use (Walker et al., 2013) over time. We have found unique effects of unaided SII, aided SII and amount of HA use on listening and language outcomes (McCreery et al., 2015; Tomblin et al., 2015), but we have not empirically tested the combined effects of these three factors on speech recognition. In pursuit of this goal, we have developed a metric we call hearing aid (HA) dosage. The concept of dosage has been applied to pharmacological and child language intervention research to study the effect of different treatment intensities (Warren et al., 2007), but it has not been utilized in the literature on childhood hearing loss. Combining HA dosage measures with longitudinal data on speech recognition in noise for children with hearing loss can inform us of the long-term effects of specific approaches to intervention and auditory access. For example, it is unclear whether higher HA dosage levels averaged across time is sufficient to support the development of speech recognition in noise, or whether fluctuations in auditory access (either due to inconsistency with wearing HAs or changes in hearing levels or aided audibility) could have a negative impact on listening in noise. The need to demonstrate the effects of aided auditory access is particularly relevant for school age children, some of whom receive less academic support in later grades (Page et al., 2018; Klein et al., 2019) and are at risk for inconsistent HA use in the classroom as they enter adolescence (Gustafson et al., 2015). Greater knowledge of the effects of HA dosage on speech recognition in noise can guide implementation of effective interventions for children with hearing loss and has the potential to motivate parents, teachers, and service providers to encourage increased HA usage.

In summary, no studies have compared developmental trajectories in speech recognition in noise between CNH and CHH. This paper describes results from a longitudinal study in which speech recognition in noise measures were collected on an annual basis in school-age CNH and CHH. The aims of this study were to: (1) compare the growth rates for speech recognition in noise for CNH and CHH, (2) determine whether CHH and CNH show similar growth rates over time, and (3) identify the auditory, cognitive, and linguistic factors that are associated with individual differences in growth rates for speech recognition in noise for CHH. It is expected that this knowledge will provide us with further insight into the everyday functional listening skills of children with and without hearing loss.

## Method

### Participants

Participants included 290 children (CHH, *n* = 199; CNH, *n* = 92) who were enrolled in a multicenter, longitudinal study on outcomes of children with mild to severe hearing loss, Outcomes of School-Age Children who are Hard of Hearing (OSACHH). The primary recruitment sites were the University of Iowa, Boys Town National Research Hospital, and University of North Carolina-Chapel Hill. Some of the children from the Iowa and Boys Town test sites also participated in a second longitudinal project that was conducted during the same time period as OSACHH. This second project was called Complex Listening in School-Age Hard of Hearing Children.

CHH had a permanent bilateral hearing loss with a better-ear four-frequency PTA in the mild to moderately severe range. One hundred seventy-nine children had a sensorineural or mixed hearing loss, 15 had a conductive hearing loss, and two had auditory neuropathy spectrum disorder. Three children did not have the type of hearing loss reported. Both CHH and CNH used spoken English as the primary communication mode and had no major vision, motor, or cognitive impairments. CNH and CHH were matched by age. There was no significant between-group difference in maternal education level [*t*(130) = −1.61, *p* = 0.11]. Demographic information, including audiologic data for the CHH, is provided in Table 1.

**Table 1:** Demographic characteristics for children who are hard of hearing (CHH) and children with normal hearing (CNH).

	CHH		CNH	
Variable	Mean (SD)	Range	Mean (SD)	Range
BEPTA (dB HL)^*^	46.16 (15.18)	7.5–90.0	<20	
Aided BESII	0.79 (0.14)	0.36–0.99	N/A	
HA dosage	10.48 (4.19)	2.56–23.91	N/A	
Age at confirmation (months)	16.75 (21.21)	0.00–92	N/A	
Age at HA fitting (months)	19.47 (21.59)	1–95	N/A	
Maternal education level (years)	15.40 (2.48)	8–22	16.05 (3.44)	8–22

*BEPTA, better-ear pure-tone average in dB hearing level (HL); BESII, better-ear speech intelligibility index; HA, hearing aid; N/A, not applicable*.

* *The criterion for study enrollment for children who were hard of hearing was BEPTA no better than 25 dB HL. Exceptions were made to include children with mild high-frequency HL (3-frequency PTA less than 25 dB HL in the better ear, but thresholds greater than 25 dB HL at 3, 4, or 6 kHz)*.

Data reported in the current analyses occurred when the children were approximately 7, 8, 9, or 10 years of age (respectively, first, second, third, or fourth grade). Children were seen for Complex Listening during first and third grade and OSACHH during second and fourth grade. All participants had completed the BKB-SIN (see description below) during at least one visit over the course of the studies.

### Procedures

This study was carried out in accordance with the recommendations of the University of Iowa Institutional Review Board, with written informed consent from all subjects. All parents of the participants gave written informed consent in accordance with the Declaration of Helsinki. The protocol was approved by the University of Iowa Institutional Review Board.

For the current analysis, participants contributed data from the BKB-SIN at up to four visits: first grade (CHH, *n* = 74; CNH, *n* = 44); second grade (CHH, *n* = 145; CNH, *n* = 79); third grade (CHH, *n* = 93; CNH, *n* = 56); and fourth grade (CHH, *n* = 128; CNH, *n* = 69). Because participants entered the study at different time points, they varied in terms of their number of visits. Furthermore, some participants missed visits between years. We had 88 CHH and CNH with one visit, 63 with two visits, 82 with three visits, and 57 with four visits.

#### Audiology Measures

Audiologic measures, HA measures, and speech recognition in noise tests were collected at every visit. For CHH, a trained clinician obtained air-conduction thresholds at 250, 500, 1,000, 2,000, 4,000, 6,000, and 8,000 Hz. Bone-conduction thresholds were obtained at 500, 1,000, 2,000, and 4,000 Hz. The four-frequency (500, 1,000, 2,000, and 4,000 Hz) better-ear pure-tone average (BEPTA) was then calculated. CNH passed a hearing screen in both ears at 20 dB HL at these four frequencies.

#### Hearing Aid Verification

At each visit, the audiologist verified that participants’ HAs were functioning appropriately. The SII (ANSI, 1997) was calculated for both ears to estimate the speech audibility based on ear canal acoustics (measured real-ear-to-coupler difference or age-average real-ear-to-coupler difference) and hearing thresholds. SII represents access of the audible speech spectrum at a conversational speech level (65 dB SPL) from a distance of 1 m. Both better-ear aided and unaided SII were calculated; CHH who did not use HAs only had unaided SII included in the analysis.

#### Hearing Aid Use

During each test visit, the caregiver completed a questionnaire related to daily HA use (available at: https://ochlstudy.org/assessment-tools). Caregivers reported average number of hours that the child wore HAs during the week and weekends, which was calculated as a weighted HA use measure [weekday use × 0.71 (5/7 days of the week) + weekend use × 0.29 (2/7 days of the week)].

#### Hearing Aid Dosage

To measure the combined effects of HA use and audibility levels (aided and unaided), we calculated a variable termed “HA dosage.” This metric can be conceptualized as how much daily access a child receives from HAs. HA dosage combines the number of hours of daily HA use with aided and unaided hearing into one weighted measure of how much auditory access a child experiences during the day^1^ from their HAs. It is calculated as HA Dosage = Daily HA Use hours^Aided Better-ear SII^ − (24 − Daily HA Use hours)^Unaided Better-ear SII^. The number of hours of daily HA use is weighted by aided SII (access to speech with HAs). If SII = 1, the child has full access to the speech spectrum for that number of hours throughout the day. The amount of time the child does not wear HAs during the day, weighted by unaided SII (access to speech without HAs), is then subtracted from the hours of use weighted by aided SII. A smaller value indicates lower HA dosage and a greater value indicates higher HA dosage.

#### Speech Recognition in Noise

We administered the BKB-SIN test (Bench et al., 1979) at each test visit. The BKB-SIN was developed to be used with children and includes short sentences with semantic and syntactic content at a first-grade reading level (Wilson et al., 2007). Recorded sentences were presented with a male talker in multi-talker background noise. The signal was calibrated at 65 dBA prior to administration. Each child received one list consisting of Part A and Part B (10 sentences per part) per visit. Lists 1–8 were administered randomly to participants; however, no participants received the same list 2 years in a row. Each sentence was presented at a different SNR, starting at 21 dB SNR and decreasing in 3 dB decrements. The tenth sentence was presented at −6 dB SNR. The test was scored in terms of the SNR needed to accurately identify 50% of the key words (i.e., SNR-50) rather than percent-correct of the total word list. Thus, a lower SNR-50 represents less difficulty understanding speech in background noise, and growth over time is seen as a downward trajectory.

#### Language Measures

Test protocols were developed to be appropriate for children utilizing spoken English in first through fourth grade. Test protocols varied depending on the year of testing. First and third grade test batteries were the same, and second and fourth grade test batteries were the same.

#### Vocabulary

At first and third grade, we administered two measures of vocabulary knowledge. The Wechsler Abbre*via*ted Scale of Intelligence-2 (WASI-2; Wechsler and Hsiao-pin, 2011) Vocabulary subtest is a standardized measure of expressive vocabulary. The examiner instructs the participant to define a series of words. Responses are scored as 0, 1, or 2 points based on the accuracy of the definition. Also at first and third grade, examiners administered the Peabody Picture Vocabulary Test-4 (PPVT-4; Dunn and Dunn, 2007). The PPVT-4 assesses receptive vocabulary; the examiner says a target word that corresponds to one of four pictures in a set, and the participant indicates the correct word. The correlation between the WASI-2 Vocabulary raw scores and the PPVT-4 raw scores was 0.81. Because the raw scores for WASI-2 Vocabulary and PPVT-4 are on different scales, we transformed each participant’s score to z-scores and averaged the z-scores together to create a single vocabulary composite score. The conversion to z-scores allowed us to standardize performance relative to our own population of participants and better measure individual growth. At second and fourth grade, we administered the Woodcock-Johnson Tests of Achievement-III Picture Vocabulary subtest (WJTA-III; Woodcock et al., 2001), which measures expressive vocabulary *via* picture naming. Again, we transformed the raw scores to z-scores so they would be on the same scale as the other vocabulary measures.

#### Working Memory

At first and third grade, we administered two standardized working memory measures from the Automated Working Memory Assessment (AWMA; Alloway, 2007). The Odd One Out subtest is a visual–spatial complex working memory span task. The participant sees three shapes in a three-square matrix on a computer screen. Two of the shapes are the same and one is different. The participant points to the shape that is the “odd one out.” The participant is then shown three empty boxes and indicates where the odd shape was located. The task is administered using a span procedure, in which the participant is asked to indicate the location of an increasing number of items. When four out of six spans within a set are identified correctly, the participant moves to the next level and the span increases by one item. The task is discontinued after three incorrect span responses within a set.

The Listening Recall subtest is a verbal complex working memory span task. The participant hears a sentence (e.g., “You eat soup with a knife”) and must determine if it is true or false. After hearing a set of two sentences, the participant repeats back the last word of each sentence in the order that he/she heard them. This task is also administered using a span procedure; if the participant accurately identifies the last words of the sentences in the correct order, the span increases by one sentence. The correlation for Listening Recall and Odd One Out raw scores for participants in first and third grade was 0.61. Raw scores were transformed into z-scores and averaged together to form a composite score.

At second and fourth grade, we administered the Listening Recall and Odd One Out tasks. In addition, we administered Backward Digit span, a working memory span measure in which the participant hears a series of numbers and is instructed to verbally repeat them back in reverse order. The correlation between raw scores for Backward Digit Span and Listening Recall was 0.50, the correlation for Backward Digit Span and Odd One Out was 0.52, and the correlation for Listening Recall and Odd One Out was 0.52 for participants in second and fourth grade. The raw scores of the three variables were transformed into z-scores and averaged together to compute a composite working memory score at second and fourth grade.

### Statistical Analyses

Our first two research questions evaluated the growth trajectories of the BKB-SIN SNR-50 scores for CHH and CNH, and whether the two groups showed similarities or differences in their rate of growth. To address these research questions, we constructed a longitudinal regression model. The fixed effects in the regression model were grade (first, second, third, and fourth); hearing status (CHH, CNH); and an interaction between grade and hearing status. To account for the correlation due to repeated measures, we included a correlation structure on the residuals. The Akaike Information Criterion (AIC; Akaike, 1974) was used to select the appropriate correlation structure within the statistical model with lower AIC values meaning better fitting models. A heterogeneous compound symmetric covariance matrix (AIC = 3315.2) was chosen over an unstructured covariance matrix (AIC = 3318.6). Therefore, the correlations between grades were approximately equal, but the variances at each time point were different.

The third research question examined which factors were associated with individual differences in growth rate for speech recognition in noise for only CHH. To construct this analysis, we used a linear regression model with a heterogeneous compound symmetric error structure to account for correlation between grades and unequal variances between grades. The dependent variable was again growth rate on BKB-SIN SNR-50 scores. The fixed effects were grade, maternal education level, age at confirmation of hearing loss, vocabulary composite z-scores, and working memory composite z-scores. Maternal education level was coded as ordinal levels (1 = High School or less, 2 = Some college, 3 = Bachelor’s degree, 4 = Post graduate, with 4 as the reference level). We also included average HA dosage across visits and change in HA dosage as separate fixed effects because HA dosage is a time-varying covariate. Change in HA dosage is calculated as each participant’s HA dosage at a given visit subtracted from the average HA dosage across visits. These separate variables allowed us to determine whether the average levels of HA dosage across visits or change in HA dosage were associated with growth rate.

## Results

### Changes in Speech Recognition in Noise Over Time

**Figure 1:**
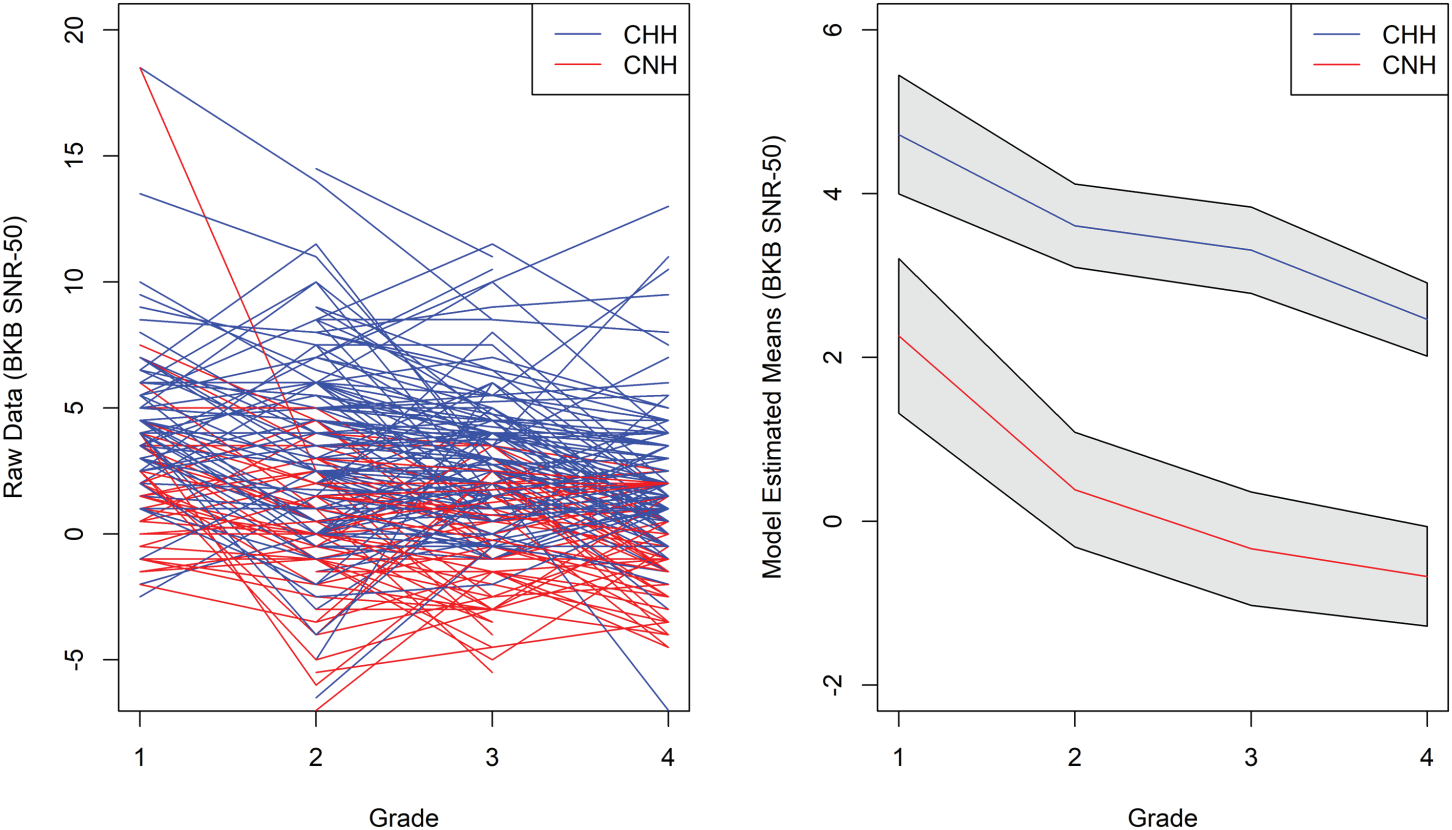
Average raw (left panel) and predicted (right panel) BKB-SIN SNR-50 scores based on model from first through fourth grade for children with normal hearing (red) and children who are hard of hearing (blue).

We found a significant main effect for grade, *F*(3, 389) = 23.78, *p* < 0.0001. Each older grade had a lower SNR-50 compared to younger grades (see Table 2). There was also a significant main effect for hearing status, *t*(284) = 8.19, *p* < 0.001. The interaction between grade and hearing status was not statistically significant, *F*(3, 389) = 1.18, *p* = 0.3154. This lack of an interaction is evident in Figure 1. On average, CHH demonstrated a SNR-50 that was 3.14 dB SNR higher than CNH, and the growth rate was consistent between groups.

**Table 2:** Summary statistics for Bamford-Kowal-Bench SNR-50 scores at each grade level.

Grade	*N*	Mean	SD	Min	Max
First	118	3.64	3.64	−2.5	18.5
Second	220	2.45	3.56	−7.0	14.5
Third	149	1.87	3.29	−5.5	11.5
Fourth	194	1.36	2.97	−7.0	13.0

*SNR, signal-to-noise ratio*.

### Factors Associated With Growth Rate in Speech Recognition in Noise for Children Who Are Hard of Hearing

**Figure 2:**
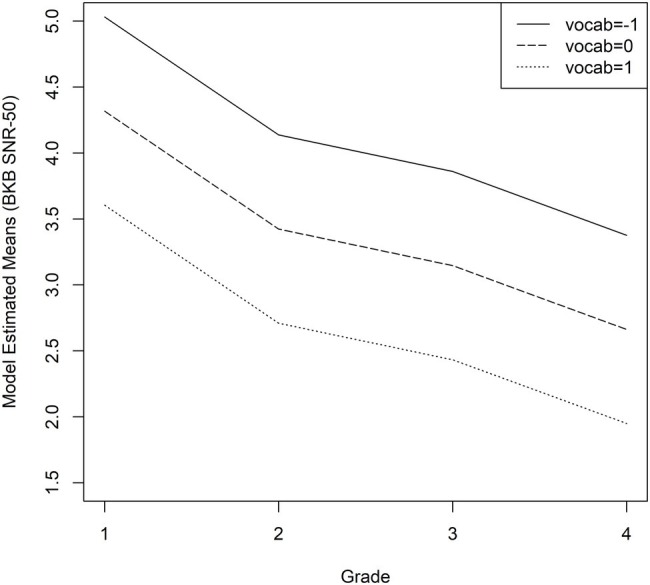
Average predicted BKB-SIN SNR-50 scores as a function of vocabulary scores for children who are hard of hearing. The solid line represents z-scores of −1 (1 SD below average), the dashed line represents z-scores of 0 (average), and the dotted line represents z-scores of +1 (1 SD above the mean).

**Figure 3:**
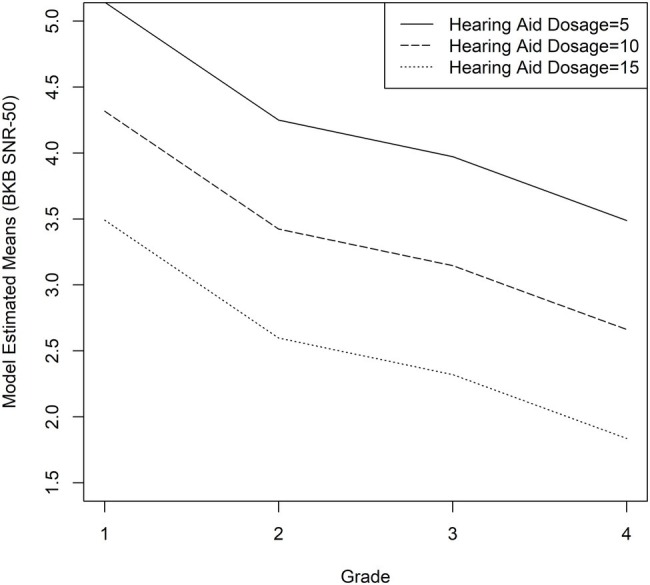
Average predicted BKB-SIN SNR-50 scores as a function of HA dosage for children who are hard of hearing. The solid line represents the 5th percentile (HA dosage = 5), the dashed line represents the 50th percentile (HA dosage = 10), and the dotted line represents the 95th percentile (HA dosage = 15).

As described in the “Statistical Analyses” section, the fixed factors were grade, maternal education level, age at confirmation of hearing loss, vocabulary composite z-score, working memory composite z-score, average HA dosage, and change in HA dosage. Interactions were not significant, so they were not included in the final model. Table 3 shows the parameters of the linear regression models. Grade level [*F*(3, 199) = 6.04, *p* = 0.0006], vocabulary composite z-scores [*F*(1, 171) = 6.00, *p* = 0.0153], and average HA dosage [*F*(1, 171) = 12.19, *p* = 0.0006] were significantly associated with rates of growth in BKB-SIN SNR-50 scores. Maternal education level [*F*(3, 171) = 0.77, *p* = 0.5098], age at confirmation of hearing loss [*F*(1, 171) = 0.04, *p* = 0.8343], working memory composite z-scores [*F*(1, 171) = 2.42, *p* = 0.1219], and change in HA dosage [*F*(1, 199) = 17, *p* = 0.6802] were not significant predictors. Stronger vocabulary skills (Figure 2), and greater average HA dosage (Figure 3) were related to better recognition of speech in noise and these patterns were consistent across age.

**Table 3:** Linear regression model with grade, maternal education level, age at confirmation of hearing loss, average vocabulary, average working memory, average HA dosage, and change in HA dosage as fixed effects and BKB-SIN SNR-50 as the dependent variable.

Parameter	Estimate	Standard error	*t* (df)	*p*
Grade (fourth grade = ref. level)				
First	1.6494	0.4192	3.93 (199)	0.0001^*^
Second	0.8767	0.3356	2.61 (199)	0.0097^*^
Third	0.3706	0.3384	1.10 (199)	0.2748
Maternal education level (post graduate = ref. level)				
High school or less	0.6879	0.6118	1.12 (171)	0.2625
Some college	−0.2326	0.5260	−0.44 (171)	0.6589
College degree	0.1497	0.4971	0.30 (171)	0.7636
Age at confirmation of hearing loss	−0.0019	0.0093	−0.21 (171)	0.8343
Average vocabulary	−0.6915	0.2822	−2.45 (171)	0.0153^*^
Average working memory	−0.4795	0.3085	−1.55 (171)	0.1219
HA dosage	−0.1761	0.0504	−3.49 (171)	0.0006^*^
Change in HA dosage	0.0373	0.0903	0.41 (199)	0.6802

*BKB-SIN, Bamford Kowal Bench Speech in Noise; SNR, signal-to-noise ratio; df, degrees of freedom*.

* *p < 0.05*.

## Discussion

The primary aim of the current study was to compare speech recognition in noise in a large group of CHH and age-matched hearing peers who have been followed on an annual basis out to fourth grade. To our knowledge, this study is among the first to track the same group of children over time and compare developmental growth rates in speech recognition for CHH compared to CNH. We also evaluated the effects of auditory access, complex working memory span, and vocabulary size on listening in noise in CHH. Identifying the mechanisms that underlie speech recognition in degraded contexts will guide clinical decision-making process for optimizing outcomes (Ching et al., 2018) and inform theories about how auditory access shapes development for CHH (Moeller and Tomblin, 2015).

### Group Differences in Growth Trajectories

Prior work on speech recognition in CHH have used cross-sectional designs with a focus on children in the 5- to 12-year-old age range (McCreery et al., 2015; Klein et al., 2017; Ching et al., 2018). CNH appear to improve in their ability to recognize words with age, reaching adult-like levels by adolescence (Eisenberg et al., 2000; Corbin et al., 2016). Based on these previous studies, we expected that CHH would have more difficulty with listening in background noise at the initial test visits and both groups would improve over time, but we were unsure of the between-group developmental patterns of these deficits. There were three possible options: (1) CHH would eventually catch up as a group to the CNH, (2) the gap in speech recognition in noise skills would widen over time, or (3) the gap would remain constant over time. Based on prior literature with adults, it seemed unlikely that CHH would catch up to their hearing peers. Results by Walker et al. (2019) provided some support for the possibility of an increasing gap in speech recognition; however, the results from the linear regression models pointed toward the third option: CHH showed a significant delay in speech recognition in noise skills at the initial visit around first grade, both groups improved in their speech recognition in noise skills over time, and the size of this gap remained approximately the same from first through fourth grade. In effect, both groups appeared to be progressing similarly with time, but the children with hearing loss started off delayed and stayed delayed. These data inform our knowledge about long-term trajectories in speech recognition in noise for children, as we do not see evidence of convergence or divergence between groups. These data also have important clinical implications because they highlight the need to continue providing support for children with all degrees of hearing loss in the general education setting as they transition from elementary grades into secondary grades. This support may take the form of resource support with a speech-language pathologist or teacher of the deaf/hard of hearing, classroom audio distribution systems, personal remote microphone systems, and/or preferential seating in the classroom.

### Individual Differences in Growth Trajectories for Children Who Are Hard of Hearing

Our second aim was to examine the factors that support growth for speech recognition in noise for CHH. Previous studies have examined age at service delivery (Sininger et al., 2010), aided audibility (Davidson and Skinner, 2006; Scollie, 2008; Stiles et al., 2012), and language (Blamey et al., 2001; Nittrouer et al., 2013) as predictive factors, but only a few have looked at the combination of auditory access, cognition, and language (McCreery et al., 2015; Klein et al., 2017; Ching et al., 2018). The findings from these previous studies have been mixed. Ching et al. found that non-verbal IQ and global language skills predicted speech recognition in noise skills for CHH, but auditory access (measured with aided SII, after controlling for unaided hearing levels) did not contribute significant variance. Klein et al. found an effect of vocabulary size, but not working memory (measured with a phonological short-term memory task) or auditory access (measured with aided SII and HA use as separate variables). McCreery et al. (2015) showed significant associations between all three factors (vocabulary size, aided SII, and phonological working memory) and word recognition in noise.

Taken together, the results of the current study may be viewed as partial support of the predictions of the ELU model. Children with stronger language skills were better able to recognize degraded speech, and children with poorer language skills had more difficulty with speech recognition in noise. Our longitudinal results indicate not just that better vocabulary skills support the ability to perceive a degraded message, but the effect of vocabulary size is stable across time. As discussed in Ching et al. (2018), these findings point toward the critical importance of language development as a focus of intervention for children with hearing loss. For some CHH who demonstrate extreme difficulty with listening in noise, this intervention may need to continue into the school age years, a time period when the intervention needs of CHH are sometimes overlooked (Antia et al., 2009). We also acknowledge that reduced auditory access in early childhood may lead to poorer speech recognition in noise skills, which in turn makes the word learning process more difficult for children with hearing loss (Walker and McGregor, 2013; Blaiser et al., 2015). We are unable to determine the direction of the relationship between vocabulary size and speech recognition noise with our current analysis approach, but future studies could employ cross-lagged analysis models or mediation analysis to infer directionality.

In contrast to the effect of vocabulary, we did not find an impact of working memory on speech recognition. The lack of an association is consistent with Magimairaj et al. (2018), and inconsistent with McCreery et al. (2015, 2017). Magimairaj and colleagues used the same clinical outcome measure, BKB-SIN, as the current study. McCreery et al. (2017) used sentences that were either syntactically correct but had no semantic meaning or had no syntactic structure or semantic meaning. Thus, the stimuli in McCreery et al. may have required children to rely on memory skills to recall the words, because they could not use linguistic bootstrapping. The BKB-SIN sentences had less of a memory load because children could use linguistic skills to remember the sentence, leading to reduced need to use working memory to repeat target words even in high levels of noise. Another possibility is that the shared variance in the vocabulary and working memory composite measures may have resulted in only vocabulary accounting for unique variance in speech recognition in noise. A larger sample size might have been able to demonstrate unique effects of both variables.

If future studies continue to support a stronger effect of language skills compared to working memory on speech recognition in children, these findings may point toward a need to modify the predictions of the ELU model. The ELU model emphasizes working memory skills as a compensatory mechanism in complex listening situations, with less focus on language skills. Because children show more variability in vocabulary breadth and depth than adults, language ability may take on a more important role in understanding distorted or masked speech, relative to working memory. Additional research is needed to test the applicability of the ELU model to the pediatric population.

In addition to cognitive and linguistic measures, we looked at how auditory access impacts individual differences in speech recognition in noise for CHH. The effects of auditory access have been inconsistent across studies (Blamey et al., 2001; Sininger et al., 2010; McCreery et al., 2015; Klein et al., 2017; Ching et al., 2018). Part of this inconsistency is due to different approaches in quantifying how much access CHH have to speech. Our measure of auditory access represents a novel approach to quantifying the HA experience of CHH. Here we developed a metric, HA dosage, that considers specific effects of amplification by weighting the amount of time children wore amplification throughout the day with aided and unaided hearing levels. The measurement of HA dosage is an improvement on previous attempts to look at auditory access in CHH because it combines sources of variability related to amplification (aided SII and HA use). It also accounts for the differential impact of HA use time based on unaided SII. When we averaged HA dosage across visits for participants, it was a significant predictor of growth rates. Like vocabulary knowledge, as HA dosage increases, CHH show better speech recognition in noise, but the patterns of change do not vary in relation to levels of HA dosage. These results highlight the need for interventions that include well-fitted HAs and consistent HA use, even in cases of mild or moderate hearing loss. While CHH with more residual hearing may perform well in quiet with or without amplification, most listening and learning situations occur in suboptimal or adverse conditions (Shield and Dockrell, 2008; Mattys et al., 2012; Ambrose et al., 2014). Increased HA dosage appears to offer some protection against the difficulties of listening in noise for these children.

We also examined whether change in HA dosage over time influenced growth rates and did not find a significant effect. CHH show variation in the consistency of auditory access during childhood (McCreery et al., 2013; Walker et al., 2013). By the school-age years, these fluctuations in auditory access do not appear to have an impact on longitudinal growth trajectories in speech recognition in noise. In addition to a lack of a significant effect for change in HA dosage, we did not find an association between speech recognition in noise with maternal education level or age at confirmation of hearing loss. Both variables have been shown to have a positive effect on auditory outcomes in children with hearing loss in previous studies (Sininger et al., 2010; McCreery et al., 2015), but the children in these earlier studies were younger than the children in the current study. Other studies with this same cohort of children indicate that CHH who receive audiologic services later demonstrate initial delays in language outcomes, but show a pattern of catching up to CHH who received services earlier by age 6 years (Tomblin et al., 2015). Thus, timing of service provision may initially affect language and listening outcomes, but the impact of age at confirmation (which is highly correlated with age at HA fitting) gradually weakens over time as other factors (vocabulary skills, aided audibility, HA use) support speech recognition in noise and ameliorate the negative effects of later confirmation of hearing loss and lower maternal education levels.

### Limitations

A strength of this study is that it is the first to document longitudinal change in growth trajectories for CNH and CHH on measures of speech recognition in noise. There are also several limitations that should be discussed. Due the study design, children were tested at different time points rather than all children participating at the same time points. This issue of inconsistent time points is a common obstacle in longitudinal research studies, as participants often start late, drop out, or skip test visits (Krueger and Tian, 2004). The use of linear mixed models for the statistical analysis accommodates data where individuals are measured at different time points (Oleson et al., 2019; Walker et al., 2019). The linear mixed model creates individual-specific trends through weighted averages of the individual observed data and the population average data so that all scores can be used in the analysis even if they are at differing time points.

Another limitation is that testing took place over a fairly limited time span (up to four visits). Further, we only tested participants up to 11 years of age, which is still a period of early adolescence. While the current data trends suggest that CNH and CHH show parallel rates of development in speech recognition in noise, it is possible that we may see differences in growth trajectories past 11 years (Corbin et al., 2016), particularly if CNH reach adult-like performance but CHH continue to improve. Future studies would need to include longitudinal data at older ages to determine if CHH eventually catch up to their hearing peers or if deficits persist with age.

We also note that the inclusionary and exclusionary criteria for this study resulted in a homogeneous cohort of children from English-speaking backgrounds with no additional motor or cognitive deficits. Thus, the current results may not generalize to linguistically diverse populations or children with hearing loss who have additional disabilities. We excluded children with profound hearing loss because we were interested in the impact of hearing loss in the mild to severe range. It is possible that we would have seen a stronger impact of age at confirmation of hearing loss if children who are deaf had been included in the sample. We did not control for the type of hearing loss because our goal was to recruit as many children with permanent hearing loss as possible; however, the majority of children presented with sensorineural hearing loss. We acknowledge that the consequences of sensorineural and conductive hearing loss can impact speech recognition in noise differently, but our limited number of children with conductive hearing loss prevents us from analyzing these children as a separate group.

A final limitation is that we restricted our speech in noise measure to the BKB-SIN test, which uses a four-talker babble as the competing signal. Other studies have shown that informational masking is increased as the number of competing talkers is decreased (Freyman et al., 2004), CNH demonstrate different developmental trajectories for two-talker maskers compared to more energetic masking signals (Corbin et al., 2016), and CHH have more difficulty with two-talker maskers than CNH (Leibold et al., 2013). We did not evaluate the effects of age, hearing status, and masker type in the present study, but this would be an important future direction in order to fully understand children’s susceptibility to background noise.

### Conclusions

The current study established longitudinal growth trajectories of speech recognition in noise for school-age CHH and CNH. As a group, CHH demonstrated deficits in speech recognition in noise. These deficits do not appear to converge toward or diverge from CNH, as the growth rates were parallel for the CHH and CNH. These findings also helped us identify the underlying mechanisms that drive growth in speech recognition, with stronger vocabulary and higher HA dosage supporting speech recognition in degraded situations.

## Data Availability Statement

The datasets generated for this study are available on request to the corresponding author.

## Author Contributions

EW and RM conceived of the presented idea. EW took the lead in writing the manuscript. CS and RM contributed to the interpretation of the result and the final version of the manuscript. JO performed the analytic and statistical calculations and data visualization. All authors provided critical feedback and helped shape the research, analysis, and manuscript.

### Conflict of Interest

The authors declare that the research was conducted in the absence of any commercial or financial relationships that could be construed as a potential conflict of interest.

## Footnotes

^1^The 24°h would include periods of time when the child is sleeping and presumably receiving little to no input. We did not ask parents how many hours their children slept on average. In the absence of those data, it seemed appropriate to calculate a full 24°h of possible input, rather than try to estimate individual sleep patterns for children.
